# Vergleich der katheterassoziierten Lebensqualität bei externen Harnableitungen: Nephrostomie vs. suprapubischer Katheter

**DOI:** 10.1007/s00120-021-01745-9

**Published:** 2022-01-13

**Authors:** A. Wiedemann, M. Weinhofer, J. Stein, C. Linné, R. Kirschner-Hermanns, A. Schorn, A. Wagner, V. Moll, U. Unger, J. Salem, T. Liebald, A. Bannowsky, S. Wirz, E. Brammen, H.-J. Heppner

**Affiliations:** 1 Urologische Abteilung, Evangelisches Krankenhaus Witten gGmbH, Pferdebachstr. 27, 58455 Witten, Deutschland; 2 Lehrstuhl für Geriatrie, Universität Witten/Herdecke, Witten, Deutschland; 3 Urologische Abteilung, Klinikum Großburgwedel, Großburgwedel, Deutschland; 4 Praxis für Urologie, Dresden, Deutschland; 5 Neuro-Urologie, Universitätsklinikum Bonn, Bonn, Deutschland; 6 Neuro-Urologie, Neurologisches Rehabilitationszentrum Bonn-Godeshöhe, Bonn-Godeshöhe, Deutschland; 7 Praxis für Urologie, Saarburg, Deutschland; 8 Praxis für Urologie, Limburgerhof, Deutschland; 9 Praxis für Urologie, Augsburg, Deutschland; 10 Praxis für Urologie, Oelsnitz, Deutschland; 11 Curos urologisches Zentrum, Abteilung für Urologie, Klinik Links vom Rhein, Köln, Deutschland; 12 Klinik für Urologie, Imland-Klinik Rendsburg, Rendsburg, Deutschland; 13 Abteilung für Anästhesiologie, Intensivmedizin, Schmerz und Palliativmedizin, Zentrum für Schmerzmedizin, Weaningzentrum, GFO-Kliniken Bonn/Cura Bad Honnef, Bonn, Deutschland; 14 Chrestos Institut, Chrestos Concept GmbH & Co. KG, Essen, Deutschland; 15 Geriatrische Abteilung und Tagesklinik, Helios-Klinikum Schwelm, Schwelm, Deutschland; 16 Institut für Biomedizin des Alterns, FAU Erlangen-Nürnberg, Nürnberg, Deutschland

**Keywords:** Katheterbezogene Lebensqualität, Nierenfistelkatheter, Suprapubischer Katheter, Palliativversorgung, Geriatrischer Patient, Catheter-associated quality of life, Percutaneous nephrostomy, Suprapubic catheter, Geriatric patients, Palliative care

## Abstract

**Einleitung:**

Die katheterbezogene Lebensqualität (LQ) bei Nephrostomieträgern wurde bisher noch nie systematisch untersucht. Dies sollte nun erstmalig vergleichend mit einer ebenfalls externen Urinableitung, dem suprapubischen Katheter, geschehen.

**Methodik:**

Das die katheterbezogene LQ untersuchende Assessment nach Mary Wilde wurde bei Patienten mit perkutaner Nephrostomie (PCN) in lebenslanger Intention und minimaler Liegedauer von 3 Monaten vorgelegt.

**Ergebnisse:**

Es zeigte sich insgesamt bei 66 Patienten (davon 42 mit unilateraler PCN) mit einem Punktwert von median 4,0 auf einer Skala von 0–5 eine nur moderat eingeschränkte katheterbezogene LQ. Diese wurde insgesamt und in allen Domänen schlechter als bei Patienten mit suprapubischem Katheter (SPK) bewertet, bei denen sich ein Score von 4,3 im Median fand. Signifikant waren die Unterschiede in den Einzelitems „Gefühl der Erniedrigung“, „Konflikte mit ärztlichem oder pflegerischem Personal“, „Angst vor schmerzhaften Katheterwechseln“, „Gefühl als kranke Person“, „Behinderungen in Aktivitäten des täglichen Lebens“ und „Besorgnis, nicht alles tun zu können, was ich mag“. Ebenso ergaben sich bei PCN-Trägern signifikant häufiger Angst vor Katheterlecks und Uringeruch. Die Anzahl der einliegenden PCN und die Grunderkrankung spielten für die Beurteilung der LQ keine Rolle.

**Schlussfolgerung:**

Erstmals wurde die katheterassoziierte LQ, die sich bei PCN-Trägern nur moderat eingeschränkt fand, mit einem validierten Assessment quantitativ eingeordnet. Die Angabe der Betroffenen, sich als „krank“ und in der Ausübung von Aktivitäten des täglichen Lebens „behindert“ zu fühlen und die Angst vor Urinleckagen und schmerzhaften Wechseln sollten Ansporn für eine sorgfältige Indikationsstellung und technisch korrekte Katheterwechsel sein.

## Abstract (en)

**Introduction:**

A percutaneous nephrostomy (PCN) affects the integrity of the patient due to the requirement of an external drainage bag and regular changes. The catheter-associated quality of life (QOL) was evaluated using a validated assessment and compared to similar patients with suprapubic bladder drainage.

**Methods:**

A validated assessment analyzing catheter-related QOL for 5 domains and 25 individual items (first published by Mary Wilde) was completed during a catheter change appointment by patients who had their unilateral or bilateral PCN for a minimum of 3 months in life-long indication.

**Results:**

In 66 patients (unilateral PCN: 42 patients), a moderate impairment of their catheter-related QOL was shown. The overall QOL score was median 4.0 on a scale of 0–5. This was rated lower—indicating a stronger impairment of QOL—than in patients with a suprapubic bladder catheter, who had a median score of 4.3. Significant differences were found related to the catheter type in the items “feeling of humiliation due to the PCN”, “conflicts with the medical or nursing staff”, “fear of painful catheter changes”, “feeling ill”, “being handicapped in activities of daily living” and “concern of not being able to do what one wants to do” and “fear of catheter leakages”. The indication for the PCN due to a malignant or benign underlying disease was not associated with the catheter-related QOL.

**Conclusion:**

For the first time, the catheter-related QOL was found to be moderately impaired in patients with a PCN using a validated assessment. Affected individuals report feeling “ill” and “limited/disabled” in activities of daily living; thus, the surgical indication should be strict. Fear of painful catheter changes and of catheter leakages indicates the need of technically correct catheter changes.

## Einleitung

Die zumeist sonographisch gestützte Einlage einer perkutanen Nephrostomie (PCN) bzw. eines Nierenfistelkatheters (NFK) wurde erstmals von Goodwin 1953 beschrieben [1]. Sie hat ihre Indikation in einem endourologisch nicht passierbaren Hindernis auf Ureterebene oder wird dann angelegt, wenn eine „innere Schienung“ mit einem Doppel-J-Katheter (DJ) z. B. bei nicht sichtbaren Harnleiterostien nicht möglich oder – etwa bei einem infizierten System – mit der Gefahr einer Septikämie verbunden wäre. Während eine Nephrostomie in einer akuten Situation z. B. bei einem infizierten Harnaufstau infolge eines Harnleitersteins nach Therapie der Ursache etwa durch eine Ureteroskopie und Infektsanierung wieder entfernt werden kann, gibt es auch palliative Situationen, in denen die Nephrostomie der lebenslangen Harnableitung – uni- oder bilateral – dient. Dies ist etwa bei einer extrinsischen Ureterkompression z. B. bei Tumoren im kleinen Becken wie Rektum‑, Uterus‑, Ovarial- aber auch Harnblasen‑, Harnleiter- oder Prostatakarzinomen der Fall. Auch narbige Harnleiterstrikturen, deren (operative) Therapie z. B. infolge ausgeprägter Komorbiditäten nicht möglich ist oder nicht gewünscht wird, gehören ebenfalls zu den Indikationen für die Einlage. Eine einliegende Nephrostomie in lebenslanger, palliativer Indikation erfordert regelmäßige Katheterwechsel in aller Regel unter Röntgenkontrolle in einer Klinikambulanz sowie regelmäßige Verbandswechsel. Diese kann der Patient wegen der Lage der PCN an der Flanke in aller Regel nicht selbst durchführen, so dass er auf Pflegepersonal oder Angehörige angewiesen ist. Zusätzlich besteht die Notwendigkeit, einen Auffangbeutel z. B. als Beinbeutel uni- oder bilateral zu tragen und regelmäßig zu wechseln. So stellt die Einlage einer Nephrostomie unabhängig und zusätzlich zur Grunderkrankung einen Eingriff in die Integrität des Betroffenen mit Verschlechterung seines Selbsthilfestatus dar. Damit einhergehend ist eine Verschlechterung der Lebensqualität (LQ) unabhängig bzw. zusätzlich von der Grunderkrankung möglich.

Umso erstaunlicher ist, dass dieser Punkt der katheterassoziierten LQ in der Literatur bisher nicht systematisch untersucht wurde. Zwar beschäftigen sich eine dreistellige Zahl von Publikationen mit technischen Aspekten der Einlage einer PCN, mit ihren Komplikationen, dem Überleben von Patienten einer Tumorentität wie z. B. dem Prostatakarzinom nach der Einlage einer Nierenfistel [2], die LQ wird jedoch nur in wenigen Arbeiten als allgemeine LQ untersucht.

So setzte eine spanische Arbeitsgruppe den „European Quality of Life 5 Dimensions“-Fragebogen bei Patienten vor Einlage einer Nephrostomie und bei dem ersten Wechsel nach 4 Wochen ein [3]. Das hier eingesetzte LQ-Assessment untersucht die allgemeinen Dimensionen Mobilität, Selbstversorgung, allgemeine Tätigkeiten, Schmerz und Angst bzw. depressive Symptome [4]. Katheterspezifische Probleme waren nicht Gegenstand der Betrachtung, so dass hier auch Einflüsse der Grunderkrankung auf die beschriebenen Veränderungen der LQ zu erwarten sind. Es kam in der genannten Untersuchung bei 150 Nephrostomieträgern zu einer statistisch signifikanten Verschlechterung der derart gemessenen LQ mit einem Abfall des LQ-Scores von 7,51 auf 5,07 Punkte – die Indikation und damit die Frage, ob es sich um eine palliative, lebenslange oder nur passagere Harnableitung handelt, wurde allerdings nicht mitgeteilt. Die Autoren wiesen auch auf eine leichte bis moderate Angst vor dem ersten Katheterwechsel bei den Betroffenen hin. Nach dem Wechsel besserte sich der entsprechende Score tendenziell, die Veränderungen waren jedoch nicht signifikant.

Monsky et al. verglichen die LQ von 45 Patienten mit einer Ureterkompression durch ein Karzinom, von denen 15 eine uni- oder bilaterale PCN und weitere 30 eine Harnleiterschiene trugen, mit einem selbst entwickelten und nicht validierten LQ-Fragebogen, der in den Dimensionen körperliches, psychisches, emotionales Wohlbefinden auch ergänzende Fragen (z. B. zu unfreiwilligem Urinverlust und Libido) enthält [5]. Es ergaben sich nach 7, 30 und 90 Tagen keine Unterschiede in der so erfragten LQ. Auch die Betrachtung der verschiedenen Formen der Harnableitung zeigte bezüglich der Art der (inneren oder äußeren) Harnableitung keine statistisch signifikanten Unterschiede. Die Autoren diskutieren, dass der Vorteil der PCN, durch die Urindrainage unter Umgehung der Harnblase keine Miktionsbeschwerden wie eine Harnleiterschiene zu verursachen, durch eine stigmatisierende äußere Ableitung mit der Notwendigkeit, einen Auffangbeutel zu tragen, egalisiert wird.

Bigum et al. verwendeten semistrukturierte Interviews zu Evaluation der LQ bei Patienten mit einer PCN [6]. Diese förderten zwar Hinweise auf ein Informationsdefizit bezüglich der Katheterableitung, Probleme in der Betreuung mit häuslichen Pflegediensten und als belastend empfundenen täglichen Umgang mit der PCN, die Ergebnisse sind jedoch aus methodischen Gründen durch das Fehlen eines systematischen und validierten Assessments eher als anekdotisch zu werten.

Es bleibt festzuhalten, dass die katheterassoziierte LQ – unabhängig von der Grunderkrankung – bisher nicht Gegenstand von Untersuchungen war. Dies erstaunt umso mehr, da im Aufklärungsgespräch eines Patienten in palliativer Situation auch und gerade die Auswirkungen auf das weitere Leben mit eventuellen Einschränkungen der LQ und Verschlechterung des Selbsthilfestatus ggf. in Abwägung zu einer Duldung einer Harnstauung unter Inkaufnahme eines eventuellen Nierenfunktionsverlustes diskutiert werden muss.

Zur Klärung der Frage der katheterassoziierten LQ wurde ein validiertes Assessment mit 25 Items erstmals von Mary Wilde publiziert und validiert [7]. Unsere Arbeitsgruppe brachte dieses Assessment bei 355 Trägern eines suprapubischen (SPK) bzw. transurethralen Blasendauerkatheters (DK) zur Anwendung [8], um die Auswirkungen der Harnblasenlangzeitdrainage insgesamt und die Unterschiede z. B. im Hinblick auf die Katheterform (transurethral/suprapubisch), das Geschlecht, die Indikation und die Dicke des Katheters zu untersuchen. Es ergab sich mit einem LQ-Score von median 4,4 auf einer Skala von 0 (maximal schlechte LQ) bis 5 (unbeeinträchtigte LQ) Punkten eine insgesamt nur mild verschlechterte LQ. Die Betrachtung der Domänenebene zeigte unterdurchschnittliche Scores bei „technischen Aspekten“ der katheterbezogenen LQ (4,0) und Kathetermanagementproblemen (4,3). Zu diesen Domänenergebnissen trugen die eine Belastung der LQ implizierenden Zustimmungs- bzw. Ablehnungswerte der Fragen nach „Angst vor Wechseln“, „Angst vor Uringeruch und Nässe“, „Sorge, dass andere Uringeruch wahrnehmen könnten“, „Angst vor größer werdenden Problemen im Alter“ bei. Erstmals konnten in dieser Untersuchung mit dem genannten Assessment geschlechterspezifische und indikationsabhängige Unterschiede bei der Akzeptanz eines Katheters gesehen werden: Während Frauen die LQ bei Einliegen eines transurethralen Katheters ähnlich wie Männer bewerteten, zeigten sich bei suprapubischer Ableitung eine signifikant schlechtere Beurteilung der LQ bei Frauen. Die Akzeptanz eines Katheters überhaupt war dann am besten, wenn die Indikation jenseits des Harntraktes gestellt wurde und nicht eine Harninkontinenz oder Blasenentleerungsstörung, sondern eine Immobilität oder Gebrechlichkeit zur Einlage eines Katheters geführt hatte. Die Akzeptanz eines SPK oder DK zeigte sich bei Patienten < 70 Jahren mit signifikant schlechteren LQ-Scores als schlechter bewertet als bei Patienten zwischen 70 und 80 Jahren bzw. > 80 Jahren (Median 3,9 vs. 4,6 vs. 4,3). Damit konnten erstmals die Auswirkungen auf die LQ durch die Katheterableitung selbst in Abhängigkeit von der Katheterform, der Katheterdicke, dem Alter und dem Geschlecht dargestellt werden. Dies könnte für den aufklärenden Arzt, der etwa die palliative Versorgung einer Harninkontinenz gegenüber einer u. U. invasiven Therapie mit dem Patienten diskutiert, hilfreiche Informationen darstellen.

### Rationale des Vergleiches der LQ bei PCN vs. SPK

Das genannte, die katheterbezogene LQ systematisch erfassende Assessment nach Mary Wilde sollte nun erstmalig bei Patienten mit einer Nephrostomie in lebenslanger Intention angewendet werden. Hierbei sollte auch geklärt werden, ob die Grunderkrankung kategorisiert in „maligne“ oder „benigne“ und eine uni- oder bilaterale Anlage einer PCN Auswirkungen auf die katheterassoziierte LQ und damit die Akzeptanz dieser Harnableitung hat. Um den Stellenwert dieser Form der Harnableitung und eventuelle Besonderheiten zu evaluieren, sollte nicht etwa ein Vergleich mit einer inneren Harnableitung per DJ-Schiene angefertigt werden, sondern erstmals die Ergebnisse dieses Assessments mit den Ergebnissen des gleichen Tools bei Patienten mit einer ebenfalls perkutanen, suprapubischen Harnblasendrainage in lebenslanger Intention verglichen werden.

Hintergrund war die Überlegung, dass eine innere Schienung per DJ-Katheter mit den häufig assoziierten irritativen Miktionsbeschwerden, mit den refluxbedingten Flankenschmerzen und den endoskopischen Katheterwechseln alle 3–6 Monate im Grunde nicht mit einer externen Ableitung mit 4‑wöchentlichen Wechseln und Röntgendurchleuchtung zu vergleichen ist. Dahingegen drainieren sowohl die PCN als auch der SPK über die Haut, sie erfordern Verbandswechsel und externe Wechselmanipulationen und ähneln sich daher mehr. Nach Auffassung der Autoren macht ein solcher Vergleich zweier perkutaner, externer Urinableitungen mehr Sinn als der Vergleich mit einer inneren Harnableitung via DJ-Katheter, bei der auf der einen Seite harnblasenassoziierte Beschwerden naturgemäß zu einer zusätzlichen Belastung führen, aber keine Behinderungen durch die externe Urinableitung bestehen. Außerdem würde ein Vergleich der katheterassoziierten LQ mit Patienten mit innerer Harnableitung eine Auswahlmöglichkeit suggerieren. Diese liegt jedoch in aller Regel nicht vor – eine perkutane Nephrostomie wird dann gelegt, wenn eine innere Schienung nicht (mehr) möglich ist. Außerdem hätte eine solche Untersuchung randomisiert vorgenommen werden müssen, dieses ist jedoch in Abhängigkeit von der Grunderkrankung nicht oder nicht in allen Fällen möglich und würde somit von Anfang an einen Bias enthalten.

## Methoden

Zur Anwendung kam das von Mary Wilde et al. in Rochester inaugurierte Assessment zur Messung der LQ von Katheterträgern in einer eigenen, professionellen Übersetzung wie schon in der Untersuchung zum Vergleich der katheterassoziierten LQ bei Trägern eines SPK bzw. DK [7]. Es handelt sich um die Adaptation eines Assessments zur Untersuchung der LQ bei Harninkontinenz [9] und wurde von der erstbeschreibenden Arbeitsgruppe an zwei Patientengruppen in ambulanter Pflege validiert. Dieses Assessment wurde nach der Übersetzung ins Deutsche und Erlangung eines positiven Ethikvotums (AZ 149/2020 der Ethikkommission der Universität Witten/Herdecke) Patienten nach Erläuterung und Unterzeichnung einer Datenschutz- und Einverständniserklärung in aller Regel im Rahmen eines Katheterwechsels durch die Autoren vorgelegt. Unabhängig von dem genannten Assessment wurde der Fragebogen wie schon in der vorangegangenen Untersuchung um eine Frage nach „mehrfachen Stürzen“ und „mehrfachen Stürzen wegen des Katheters“ ergänzt, um eventuelle Einflüsse der Harnableitung auf das geriatrische Syndrom „Sturzneigung“ zu untersuchen. Der im Originalassessment enthaltene Fragenkomplex nach Schleimhautirritationen im Intimbereich wurde in der Version für diese Erhebung (weil, da der NFK den Flankenbereich tangiert, als nicht zutreffend) gestrichen. Haupteinschlusskriterium war das Einliegen einer unilateralen oder bilateralen PCN für mindestens 3 Monate in lebenslanger, palliativer Indikation. Es erfolgte die Auswertung der demographischen Daten sowie die strukturierte Darstellung der globalen LQ und der in den einzelnen, vom Assessment vorgegebenen Domänen und Einzelitems analog der Untersuchung der LQ bei Trägern eines transurethralen oder suprapubischen Blasenkatheters. Die Ergebnisse bezogen auf die Träger eines NFK wurden denen des suprapubischen Blasenkatheters gegenübergestellt. Dabei erfolgte eine Deskription der Patientendaten bei metrischen, annähernd normalverteilten Variablen mit Hilfe des Mittelwertes und der Standardabweichung; schiefverteilte Variablen wurden mit Hilfe des robusteren Medians und dem Interquartilsabstand dargestellt. Die Bewertungen der einzelnen Fragen des Assessments wurden mit ihrem absoluten und relativen Vorkommen beschrieben und mit Hilfe des χ^2^-Unabhängigkeitstest verglichen.

## Ergebnisse

Ein vollständiges LQ-Assessment lag bei 66 Patienten mit NFK vor, die sich zwischen April 2020 und Februar 2021 in den Kliniken bzw. Ambulanzen der Autoren vorgestellt hatten. Bei 64 Patienten lag die Angabe, ob eine singuläre oder bilterale PCN einlag, vor. 42 Patienten (65,6 %) trugen dabei eine unilaterale, 22 (34,4 %) bilaterale PCN. Die Indikation für die Nephrostomieanlage bestand bei 34 Patienten in einer karzinombedingten Ureterkompression, bei 31 Patienten lag eine benigne Grunderkrankung vor (52,3 vs. 47,7 %, bei einem Patienten fehlende Angabe). Die Indikationen zur PCN bestanden im Einzelnen aus einem Harnblasentumor (*n* = 14), einem Harnleitertumor (*n* = 2), einem Uteruskarzinom (*n* = 8), einem Prostatakarzinom (*n* = 5), kolorektalen Karzinomen (*n* = 4) und Mammakarzinomen (*n* = 2). Weitere Indikationen für die Nierenfistelung stellten radiogene Harnleiterengen (*n* = 4), ein Harnleitertrauma (*n* = 3), ein Ormond-Syndrom (*n* = 1), eine Schrumpfblase (*n* = 1), eine vesikovaginale Fistel (*n* = 1) und eine narbige postoperative Harnleiterenge (*n* = 4) dar. In 19 Fällen war die Ursache für die bestehende Harnleiterstriktur unklar (Mehrfachnennungen möglich). Patienten mit einem Tumorleiden trugen ihre PCN zum Zeitpunkt der Erhebung im Mittel 20 Monate, Patienten mit benigner Grunderkrankung im Mittel 27 Monate. Es kamen bei 55 Patienten (90,2 %) ein NFK von ≤ 14 Charr., bei weiteren 2 Patienten ein NFK von 16 Charr. (3,3 %) und bei 4 Patienten ein NFK von ≥ 18 Charr. zur Anwendung (Rest: keine Angaben). Größere oder kleinere Katheterdurchmesser fanden in der untersuchten Patientengruppe keine Anwendung.

### Vergleich der LQ bei Trägern eines NFK im Vergleich mit Trägern eines SPK

**Figure 1:**
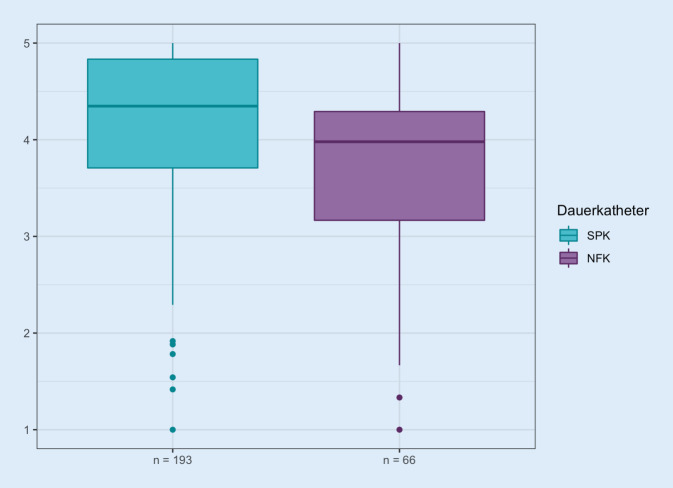

Die globale katheterassoziierte LQ wurde von Trägern eines NFK mit median 4,0 Punkten (5 Punkte würden einer nicht beeinträchtigten, 0 Punkte einer maximal beeinträchtigten LQ entsprechen) gemessen. Damit liegt ein niedrigerer Gesamtscore im Vergleich mit Trägern eines suprapubischen Katheters vor, der hier median 4,3 betragen hatte (s. Abb. 1).

Dieser Trend zeigt sich auch bei der Betrachtung aller 4 Einzeldomänen (s. Tab. 1).

**Table 1:** 

Domäne	LQ-Score bei Trägern einer PCN (*Median*; Mittelwert)	LQ-Score bei Trägern eines SPK (*Median*; Mittelwert)
D09 (Kathetermanagement-Probleme)	*3,9*; MW 3,6 ± 1,1	*4,4*, MW 4,1 ± 0,9
D10 (interpersonelle Probleme)	*4,3*; MW 3,9 ± 1,1	*4,7*; MW 4,4 ± 0,8
D11 (psychosoziale Probleme)	*3,8*; MW 3,7 ± 1,1	*4,8*; MW 4,7 ± 1,1
D12 (allgemeine LQ)	*3,7*; MW 3,5 ± 1,1	*4,3*; MW 3,9 ± 1,2

*LQ* Lebensqualität, *MW* Mittelwert

Die Betrachtung der LQ heruntergebrochen auf die Einzelitems des Assessments zeigt für die Harnblasendrainage per SPK im Vergleich mit der PCN folgendes Bild (s. Tab. 2).

**Table 2:** 

Domäne	Frage		*n* =	Median	Mittelwert	SD	IQR
*09 Kathetermanagementprobleme* (LQDS median NFK: 4,4 SPK: 3,9)	„Ich bin besorgt, wegen einer Katheterundichtigkeit nass zu werden“ (9a)	SPK	190	5	3,92	1,47	2,0
		NFK	65	4	3,46	1,67	3,0
	„Ich bin besorgt, in der Nähe eine Toilette zu finden“ (9b)	SPK	188	5	4,41	1,16	1,00
		NFK	63	5	3,98	1,40	2,00
	„Ich bin besorgt, öffentliche Toiletten zu finden, die geeignet sind, dass ich dort den Katheterbeutel leeren kann“ (9c)	SPK	184	5	4,45	1,13	0,25
		NFK	64	4	3,75	1,54	2,25
	„Ich bin besorgt über den Katheterblock“ (9d)	SPK	178	5	4,26	1,26	1,0
		NFK	62	5	3,94	1,40	2,0
	„Ich bin besorgt, dass andere den Uringeruch an mir wahrnehmen könnten“ (9e)	SPK	186	5	3,89	1,41	2,0
		NFK	65	4	3,55	1,58	3,0
	„Ich bin besorgt, dass die Katheterprobleme größer werden könnten, wenn ich älter werde“ (9f)	SPK	187	5	3,96	1,37	2,0
		NFK	66	4	3,44	1,49	3,0
	„Ich muss darauf achten, was ich trinke“ (9g)	SPK	190	5	3,91	1,70	3,0
		NFK	65	4	3,45	1,57	3,0
	„Ich bin besorgt, eine Harnwegsinfektion zu bekommen“ (9h)	SPK	188	4	3,52	1,57	3,0
		NFK	66	3	3,06	1,47	2,0
	„Ich bin besorgt, den Katheterbeutel leeren zu können, bevor er zu voll wird“ (9i)	SPK	181	5	4,33	1,22	1,0
		NFK	65	5	3,89	1,43	2,0
	„Ich fühle mich erniedrigt durch den Katheter“ (9j)	SPK	189	5	**4,3**	1,18	1,0
		NFK	65	4	**3,69**	1,41	2,0
*10 Interpersonelle Probleme* (LQDS median SPK: 4,7 NFK 4,3)	„Ich bin besorgt, meine Betreuer über die richtige Katheterpflege zu informieren“ (10a)	SPK	186	5	4,56	0,95	0,0
		NFK	82	5	4,35	1,24	0,75
	„Ich bin besorgt, Ärzte und Pflegepersonal über das Problem der ‚autonomen Dysreflexie‘ zu informieren“ (10b)	SPK	182	5	4,53	1,03	0,0
		NFK	60	5	4,18	1,33	1,0
	„Ich bin besorgt über mögliche Konflikte mit Ärzten und Pflegepersonal in Bezug auf den Katheter“ (10c)	SPK	186	5	**4,61**	0,9	0,0
		NFK	65	5	**4,18**	1,36	1,0
	„Ich hatte eine schwere Zeit wegen Katheterschmerzen“ (10d)	SPK	188	5	**4,18**	1,32	1,0
		NFK	66	4	**3,77**	1,45	2,0
	„Ich bin besorgt, wie der Katheter meine Sexualität beeinflussen könnte“ (10e)	SPK	183	5	4,55	1,11	0,0
		NFK	64	5	4,12	1,43	2,0
	„Mein Katheter begrenzt die Auswahl meiner Kleidung“ (10f)	SPK	188	5	4,14	1,36	1,0
		NFK	65	4	3,20	1,66	4,0
*11 Psychosoziale Probleme* (LQDS median SPK: 4,8 NFK: 3,8)	„Der Katheter bewirkt, dass ich mich als kranke Person fühle“ (11a)	SPK	190	5	**4,07**	1,37	2,0
		NFK	62	4	**3,68**	1,42	2,0
	„Ich kann mein Leben wegen des Katheters weniger genießen“ (11b)	SPK	190	5	**4,0**	1,39	2,0
		NFK	63	4	**3,27**	1,54	3,0
	„Ich bin darüber frustriert, dass der Katheter mich davon abhält, zu tun, was ich mag“ (11c)	SPK	191	5	**4,03**	1,41	2,0
		NFK	61	4	**3,41**	1,48	3,0
	„Der Katheter führt bei mir zu einem Gefühl der Hilflosigkeit“ (11d)	SPK	190	5	4,35	1,19	1,0
		NFK	63	5	4,10	1,29	1,0
	„Ich habe das Gefühl, dass ich mein Zuhause nicht mehr für längere Zeit verlassen kann“ (11e)	SPK	191	5	4,44	1,13	1,0
		NFK	62	5	4,06	1,30	1,0
*12 Katheterbezogene LQ* (LQDS median SPK: 4,3 NFK: 3,7)	„Ich bin besorgt über mögliche Katheterlecks“ (12a)	SPK	188	5	**3,84**	1,46	2,0
		NFK	62	4	**3,48**	1,35	2,75
	„Ich bin besorgt über einen unfreiwilligen Urinverlust“ (12b)	SPK	189	5	3,95	1,41	2,0
		NFK	62	4	3,66	1,40	2,0
	„Ich bin besorgt über möglicherweise schmerzhafte Katheterwechsel“ (12c)	SPK	192	5	**3,95**	1,43	2,0
		NFK	63	3	**3,40**	1,42	3,0

*Fett markiert* sind die Einzelergebnisse, die im χ^2^-Test eine Abhängigkeit vom Kathetertyp „NFK“ vs. „SPK“ zeigen (*LQDS*, Lebensqualitätsdomänenscore im Median)

*SPK* suprapubischer Blasenfistelkatheter, *NFK* Nierenfistelkatheter, *n* Anzahl der Beobachtungen, *MW* Mittelwert, *SD* Standardabweichung, *IQR* Interquartilsabstand, *LQ* Lebensqualität

Die Betrachtung der Einzelfragen zeigt im χ^2^-Test eine Abhängigkeit der Fragestellung vom Kathetertyp (Vergleich SPK vs. NFK) bei den Einzelitems 9J („ich fühle mich durch den Katheter erniedrigt“, *p* = 0,007), 10c („ich bin besorgt über mögliche Konflikte mit Ärzten und Pflegepersonal in Bezug auf den Katheter“, *p* = 0,04), 10d („ich hatte eine schwere Zeit wegen Katheterschmerzen“, *p* = 0,009), 11a („der Katheter bewirkt, dass ich mich als kranke Person fühle“, *p* = 0,008), 11b („ich kann mein Leben wegen des Katheters weniger genießen“, *p* = 0,006), 11c („ich bin darüber frustriert, dass der Katheter mich davon abhält, zu tun, was ich mag“, *p* = 0,002), 12a („ich bin besorgt über mögliche Katheterlecks“, *p* = 0,023) und 12c („ich bin besorgt über schmerzhafte Katheterwechsel“, *p* = 0,002) mit der Tendenz (vgl. Ergebnisse aus Tab. 2), dass mehr Patienten mit einem NFK als mit einem SPK eine Einschränkung der LQ in diesen Punkten durch eine Zustimmung zur jeweiligen Aussage angeben. Wie in der Untersuchung der verschiedenen Formen der Harnblasenlangzeitdrainage wurde die Frage nach Stürzen („mehr als einmal im letzten Jahr gestürzt“ und „mehr als einmal wegen des Katheters im letzten Jahr gestürzt“) gestellt. Es zeigte sich mit 19,2 % bzw. 8,3 % positiver Antworten bei NFK-Trägern eine höhere Sturzinzidenz i. Allg. und katheterbedingt als bei SPK-Trägern (14,2 % bzw. 1,1 %). Während die Unterschiede für mehrfache Stürze zwischen Trägern eines NFK und eines SPK sich insgesamt im Fischer-Test mit einem *p* = 0,387 als nicht signifikant erwiesen, war die Angabe von mehrfachen Stürzen wegen des Katheters bei Patienten mit einer PCN signifikant häufiger (*p* = 0,01).

### Unterschiede in der LQ abhängig von der Indikation

In einem zweiten Teil der Untersuchung sollte der Frage nachgegangen werden, ob ein Malignom als Grund für die Harnstauung und die Indikation für die NFK-Anlage einen Einfluss auf die Beurteilung der LQ haben könnte. Bei Frauen mit NFK (*n* = 33) lag bei 20 oder 64,5 % der Fälle ein Malignom vor, während dies bei Männern mit NFK (*n* = 32) nur bei 11 Fällen oder 35,5 % der Fall war. Die Unterschiede waren nicht signifikant (*p* = 0,062). Patienten mit einer malignen Grunderkrankung wiesen im Vergleich mit solchen mit einer benignen Ursache der Ureterkompression eine kürzere Liegezeit des NFK auf (20 vs. 27 Monate). Sie trugen allerdings nicht häufiger eine beidseitige PCN (benigne Indikation: 12 Patienten unilaterale PCN oder 35,3 %, maligne Indikation: 10 Patienten unilaterale PCN oder 33,3 %). Die Bewertung der globalen LQ zeigte für Patienten mit malignombedingt einliegender PCN gegenüber nicht-malignombedingt einliegender PCN nur eine geringgradig schlechter bewertete LQ (Median 3,9 vs. 4,0, MW 3,7 ± 0,9 bzw. 3,7 ± 0,8). Die Auswertung der Beurteilung der LQ auf Domänenebene und bezogen auf die Einzelitems ergab nur in einem Fall statistisch signifikante Unterschiede zwischen Patienten mit einem Malignom oder einer gutartigen Erkrankung als Ursache für die zur Nephrostomieanlage führenden Ureterkompression. Die Aussage 12a („ich bin besorgt wegen Katheterlecks“) zeigt eine signifikante Abhängigkeit zum Tumorleiden (*p* = 0,019). Der Vergleich der Mediane zeigt, dass Patienten mit Tumorleiden dieser Aussage häufiger zustimmten als bei Patienten ohne maligne Grunderkrankung. Die Frage nach mehrfachen Stürzen allgemein bzw. wegen des NFK zeigte keine statistisch signifikanten Unterschiede zwischen diesen beiden Patientengruppen.

### Abhängigkeit der katheterassoziierten LQ von dem Vorhandensein eines singulären oder beidseitigen NFK

Insgesamt wurde bei 64 Patienten die Anzahl der einliegenden NFK (unilateral/bilateral) dokumentiert. Es trugen 52,4 % der Männer und 47,6 % der Frauen einen einseitigen NFK; entsprechend 45,5 % bzw. 54,5 % einen bilateralen NFK (Unterschiede n. s.). Auch die Altersverteilung und die Kathetergrößen und die Verteilung im Hinblick auf eine benigne oder maligne Indikation zeigten keinen signifikanten Zusammenhang zu der Anzahl der einliegenden NFK. Auch die Liegedauer der Nephrostomie war für einseitige NFK mit im Mittel 20 Monaten nicht signifikant unterschiedlich zu der beidseitiger Nephrostomien mit 24 Monaten (*p* = 0,308). Gleiches gilt für die Indikation zur PCN, die in 52,4 bei einseitiger bzw. 54,5 % bei beidseitiger Nephrostomie einem Malignom zuzuordnen war.

**Figure 2:**
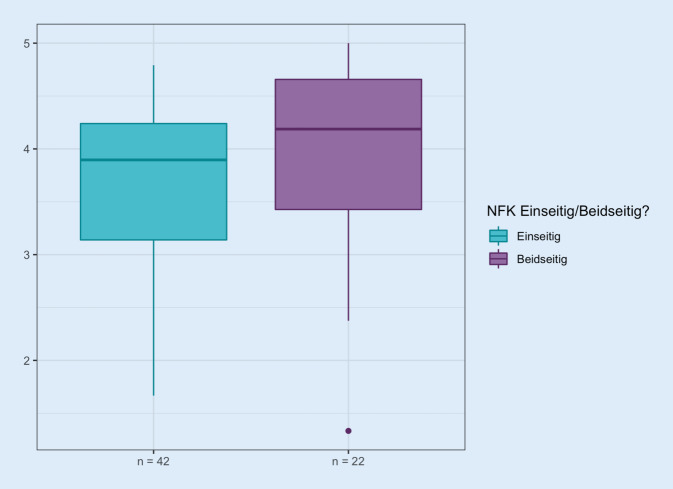

Die LQ wurde bei Trägern eines einseitigen NFK mit einem Median von 3,9 (SD 0,8) gegenüber der bei Trägern eines beidseitigen NFK mit 4,2 (SD 1,0) insgesamt schlechter bewertet (n. s., s. Abb. 2).

**Figure 3:**
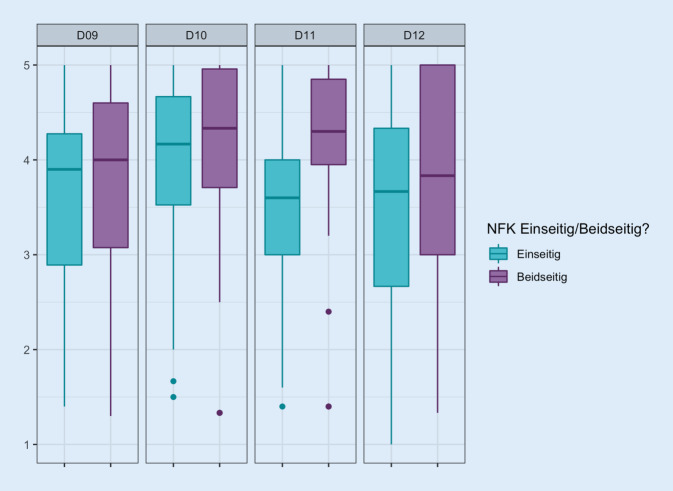

Heruntergebrochen auf die Domänenebene zeigte sich im Wilcoxon-Rangsummentest ein signifikanter Unterschied (*p* = 0,016) in der Domäne 11 „psychosoziale Probleme“ (s. Abb. 3); die Betrachtung der Einzelfragen ergab in keinem Fall signifikante Unterschiede zwischen Trägern eines einseitigen bzw. beidseitigen NFK.

## Diskussion

Die Einlage einer PCN in lebenslanger Indikation stellt durch die Notwendigkeit regelmäßiger Wechsel unter Röntgenkontrolle und der Erfordernis des Tragens eines externen Ableitungssystems sowie regelmäßiger Verbandswechsel durch Angehörige oder einen Pflegedienst einen fundamentalen Eingriff in die körperliche Integrität des Patienten dar. Sie geht mit einem über die Grunderkrankung hinausgehenden Autonomieverlust und Einschränkung des Selbsthilfestatus einher und macht in aller Regel 4‑wöchige Wechsel unter Durchleuchtung und damit in aller Regel Krankenhaus- bzw. Ambulanzkontakte nötig, die ggf. mit Taxi- oder Krankentransporten verbunden sind und nicht im Rahmen eines Hausbesuchs abgewickelt werden können. Diese Umstände sind dann in Kauf zu nehmen, wenn eine Therapie der Grunderkrankung nicht mehr möglich ist, mit hohen Risiken verbunden ist oder vom Patienten nicht gewünscht wird. Als Beispiele hierfür gelten z. B. die Harnstauungsniere infolge einer Implantationsenge bei Zustand nach Anlage einer Neoblase oder die chronische Stauungsniere infolge einer extrinsischen Ureterkompression z. B. durch Lymphome eines Malignoms im kleinen Becken oder als narbige Enge nach Bestrahlung. Im Einzelfall müssen mit dem Patienten die Alternativen etwa wie ein extraanatomischer pyelovesikaler Bypass, ein Ureterersatz oder -repair besprochen werden. In diese Diskussion spielen bei der Abwägung des Für und Wider eine Reihe von Faktoren wie die Prognose der Grunderkrankung, die Lebenserwartung des Patienten, die verbleibende Nierenfunktion und letztlich auch der Patientenwunsch, seine Komorbiditäten, seine Mobilität und Gebrechlichkeit und die Erfolgsaussichten eines solchen Eingriffs im Hinblick auf eine problemlose Urindrainage hinein. Bei dieser Betrachtung fehlen bisher vollkommen systematische Informationen über die Auswirkungen dieser Form der Harnableitung auf die LQ unabhängig von der Grunderkrankung. Naturgemäß kann im Einzelfall die globale LQ auch durch die Grunderkrankung mitbeeinflusst werden – Aspekte wie Besorgnis vor Katheterwechseln, Angst vor unfreiwilligem Urinverlust mit Uringeruch, Behinderungen bei der Auswahl von Kleidung, Angst vor Harnwegsinfekten könnten jedoch das tägliche Leben weit mehr beeinflussen als die Grunderkrankung selbst und damit zu einer (weiteren) Verschlechterung der LQ beitragen. Systematische Untersuchungen zur katheterassoziierten LQ bei PCN-Trägern fehlten bisher vollständig.

So entstand im Arbeitskreis „geriatrische Urologie“ der DGU die Idee, das einzige bisher existierende validierte Assessment zur Messung der katheterbezogenen Aspekte der LQ auf Patienten mit NFK in lebenslanger Indikation anzuwenden.

Dieses Assessment war erstmals durch den genannten Arbeitskreis an einer größeren Patientengruppe mit SPK und DK vergleichend untersucht worden. In dieser Untersuchung konnte so gesichert werden, dass z. B. bei Frauen mit Harninkontinenz als Indikation für einen SPK die katheterassoziierte LQ signifikant schlechter ist als bei Männern mit SPK – am ehesten einem persistierenden transurethralen Urinverlust geschuldet. Die geringste Einschränkung der katheterassoziierten LQ wurde dann angegeben, wenn die Indikation in einer Immobilität oder Gebrechlichkeit bestand und der Patient über 80 Jahre alt war. Überhaupt zeigt die erste Messung der katheterassoziierten LQ mit einer nur milden Einschränkung der globalen LQ, dass die Wahl einer transurethralen oder suprapubischen Harnableitung als Palliativversorgung nicht automatisch mit einer dramatischen Verschlechterung der LQ einhergeht. Die Autoren dieser Untersuchungen bewerteten diese Informationen als außerordentlich hilfreich in der (Differential)indikation einer palliativen Katheterableitung der Harnblase.

Das schon erwähnte Assessment der katheterbezogenen LQ von Mary Wilde sollte nun auf Patienten mit einer PCN in lebenslanger Indikation übertragen werden. Die Idee entstand, hier bewusst keinen Vergleich mit einer inneren Harnleiterschienung anzustellen, wie er bereits mehrfach publiziert wurde [10–12], sondern die PCN mit dem SPK zu vergleichen. In einer palliativen Situation für eine Harnableitung einer Stauungsniere in lebenslanger Indikation stellt sich die Alternative zwischen einer inneren und äußeren Harnableitung häufig nicht; diese beiden Formen der Harnableitung sind schon wegen ihrer Begleitumstände (Fremdkörper und Drainagesystem extern, Verbandswechsel, hochfrequente, externe Wechsel) nicht gut zu vergleichen. Im Zweifelsfall wird jeder Therapeut und Patient zu der inneren, weniger stigmatisierenden Harnableitung neigen, zumal DJ-bedingte Miktionsbeschwerden etwa durch Antimuskarinika oder der Wahl besonderer DJ-Modelle bzw. -Materialien wenigstens partiell behandelbar erscheinen. Daher sollten hier die Ergebnisse des Assessments bei PCN-Patienten mit denen mit einem SPK aus der vorangegangenen Untersuchung verglichen werden.

### Beurteilung der LQ insgesamt

Die Ergebnisse für PCN-Patienten zeigen, dass der globale LQ-Score bei PCN-Patienten mit median 4,0 vs. 4,3 eine stärkere Belastung als bei Patienten mit SPK ausweist. Insgesamt ist die LQ mit 4,0 auf einer Skala von 0 (maximal beeinträchtigte LQ) bis 5 (keine Beeinträchtigung) nur moderat eingeschränkt. Die stärkere Beeinträchtigung der katheterassoziierten LQ bei PCN-Patienten spiegelt sich in niedrigeren Scores in allen 4 untersuchten Domänen wider (s. Tab. 1). Insbesondere zeigt sich in der statistischen Analyse eine Abhängigkeit vom Kathetertyp „NFK“ vs. „SPK“ mit der Angabe einer höheren Zustimmung bei der Angabe eines Gefühls einer Erniedrigung durch den Katheter, bei der Mitteilung von Konflikten mit ärztlichem und pflegerischem Personal und bei dem Durchleben von Katheterschmerzen. Auch geben Patienten mit PCN statistisch signifikant häufiger an, sich als kranke Person zu fühlen, durch die PCN von Aktivitäten des täglichen Lebens abgehalten zu werden und nicht tun zu können, „was man mag“. Zusätzlich wird statistisch signifikant häufiger eine Besorgnis vor Katheterlecks und schmerzhaften Katheterwechseln angegeben. Damit zeigt sich – erstmals durch ein validiertes Assessment abgesichert – das Ausmaß der Beeinträchtigung der katheterassoziierten LQ bei PCN-Trägern. Gründe hierfür könnten die anatomischen Bedingungen bzw. Unterschiede sein. Liegt der SPK in einem Hohlorgan mit in aller Regel größerer Kapazität, bring die Katheterableitung des Nierenbeckens mit der gewöhnlich nur wenigen Milliliter betragenden Kapazität und der typischen, „zerklüfteten“ Konfiguration mit den Nierenkelchen besondere Probleme mit sich: Jedem Urologen bekannt sind hier Fehllagen im Nierenkelch oder Kelchhals, die zu einer inkompletten Drainage, zu Abflussstörungen mit Schmerzen oder parastomalen Urinabgängen führen.

### Einflussfaktor Dignität der Grunderkrankung

In diesem Kontext spielt die Indikation für die PCN – stratifiziert nach „tumorbedingte Indikation“ vs. „benigne Indikation“ für die Beurteilung der LQ durch die Patienten keine Rolle – weder in der Angabe des globalen LQ-Scores, noch auf Domänen- oder Einzelitemebene. Die statistische Analyse zeigte lediglich bei einem einzigen Item (12a, „Sorge vor Katheterlecks“) einen statistisch signifikant schlechteren Wert bei Tumorpatienten. Dies deutet daraufhin, dass es vor allem „technische“ Aspekte sind, die zu den Patienten belastenden Problemen und damit zu einer Einschränkung der LQ in seiner Beurteilung führen.

### Einflussfaktor Alter

Zu diskutieren ist, ob das Alter als Einflussfaktor für die Akzeptanz eine Rolle spielt. So hatte der Vergleich des DK mit dem SPK höhere LQ-Scores bei Patienten > 80 Jahre als möglichen Hinweis auf einen solchen Effekt gezeigt. In dieser Untersuchung lag das mittlere Alter aller Patienten mit Blasenkatheter bei 76,5 ± 12,2 Jahre, das der Träger eines SPK 74,4 ± 12,6 Jahre, eines DK 78,9 ± 11,1 Jahre. Damit ist jedoch der Unterschied des mittleren Alters zu PCN-Patienten mit 72,8 ± 12,6 Jahren in benigner Indikation bzw. 73,5 ± 14,8 Jahren mit Tumorleiden nur marginal unterschiedlich, so dass dieser Effekt als Erklärung untauglich sein dürfte.

### Einflussfaktor ein- oder beidseitige Nephrostomie

Ein anderer Punkt könnte jedoch für die schlechtere Beurteilung der LQ bei PCN-Trägern verantwortlich sein: knapp die Hälfte der Patienten (*n* = 22) ist Träger einer beidseitigen PCN – was naturgemäß bei Patienten mit SPK nicht der Fall ist. So könnte allein dieser Umstand der Belastung bzw. Behinderung durch eine beidseitige PCN mit entsprechender Doppelbehinderung bzw. doppeltem Risiko von Urinlecks, Geruch von austretendem Urin oder schmerzhaften Katheterwechseln für den beschriebenen Effekt verantwortlich sein. Umso überraschender ist der Befund, dass Patienten mit unilateralem NFK ihre LQ schlechter einstufen, als es Patienten mit bilateralem Katheter tun. Hier zeigen sich signifikante Unterschiede in der Beurteilung der LQ in der Domäne 11, die „intrapsychische Probleme“ mit den Fragen „Der Katheter bewirkt, dass ich mich als kranke Person fühle“ (11a), „Ich kann mein Leben wegen des Katheters weniger genießen“ (11b), „Ich bin darüber frustriert, dass der Katheter mich davon abhält, zu tun, was ich mag“ (11c), „Der Katheter führt bei mir zu einem Gefühl der Hilflosigkeit“ (11d) und „Ich habe das Gefühl, dass ich mein Zuhause nicht mehr für längere Zeit verlassen kann“ (11e) abfragt. Weder die Liegedauer noch die Indikation für die Nephrostomie im Hinblick auf ein Malignom oder eine benigne Grunderkrankung unterschieden sich bei dem Vergleich zwischen einseitiger und beidseitiger PCN. Möglicherweise spielt hier die Krankheitsverarbeitung für die Akzeptanz der PCN eine Rolle: während sie bei einer beidseitigen Ableitung per PCN vom Patienten als alternativlos akzeptiert wird, lässt die unilaterale Ableitung eine solche intrapsychische Verarbeitung durch den stetigen Vergleich mit der „gesunden“ oder „innerlich geschienten“ Seite nicht oder nur schlechter zu.

Insgesamt nimmt besonders die Angabe einer Beeinträchtigung der LQ durch „technische Katheterprobleme“ wie einen okklusionsbedingen Urinaustritt mit Geruchsentwicklung oder Besorgnis vor schmerzhaften Katheterwechseln den behandelnden Urologen in die Pflicht, auch in diesen für den Patienten wichtigen Punkten durch handwerkliche Sorgfalt etwa durch korrektes Positionieren der PCN im Hohlsystem, adäquates Befüllen des Ballons und sorgfältige Verbandswechsel diese Probleme zu minimieren. Da offenbar tendenziell die beidseitige PCN eine bessere Akzeptanz dieser Form der Harnableitung als eine unilaterale besitzt, bedeutet dies auch, besonders bei einer unilateralen Harntransportstörung nach einer die Nephrostomie vermeidenden Lösung zu suchen.

## Fazit für die Praxis

- Zwar ist die katheterassoziierte Lebensqualität (LQ) bei Trägern einer perkutanen Nephrostomie (PCN) mit median 4,0 auf einer Skala von 0–5 nur moderat eingeschränkt, sie wird aber insgesamt schlechter bewertet als von Trägern eines suprapubischen Katheters (SPK) mit median 4,3 Punkten.
- Insbesondere die signifikant schlechter als der Durchschnitt bewerteten Einzelitems „Angst vor schmerzhaften Katheterwechseln, Katheterlecks und Uringeruch“ nehmen den die PCN wechselnden Urologen in die Pflicht, für schmerzarme und technisch korrekte Katheterwechsel zu sorgen.
- Erstmalig ist damit unter Berücksichtigung der gefundenen Daten eine Diskussion „palliative Urindrainage vs. Harnleiterrepair“ in Kenntnis der Auswirkungen einer Nephrostomie als Dauerableitung gemessen mit einem validierten, speziellen Assessment möglich.
